# Exploiting Pan Influenza A and Pan Influenza B Pseudotype Libraries for Efficient Vaccine Antigen Selection

**DOI:** 10.3390/vaccines9070741

**Published:** 2021-07-05

**Authors:** Joanne Marie M. Del Rosario, Kelly A. S. da Costa, Benedikt Asbach, Francesca Ferrara, Matteo Ferrari, David A. Wells, Gurdip Singh Mann, Veronica O. Ameh, Claude T. Sabeta, Ashley C. Banyard, Rebecca Kinsley, Simon D. Scott, Ralf Wagner, Jonathan L. Heeney, George W. Carnell, Nigel J. Temperton

**Affiliations:** 1 Viral Pseudotype Unit, Medway School of Pharmacy, The Universities of Greenwich and Kent at Medway, Chatham ME4 4BF, UK; J.M.Del-Rosario@kent.ac.uk (J.M.M.D.R.); k.da-costa@kent.ac.uk (K.A.S.d.C.); francesca.ferrara@stjude.org (F.F.); g.s.mann@kent.ac.uk (G.S.M.); S.D.Scott@kent.ac.uk (S.D.S.); 2 Department of Physical Sciences and Mathematics, College of Arts and Sciences, University of the Philippines Manila, Manila 1000, Philippines; 3 DIOSynVax, Cambridge CB3 0ES, UK; matteo@diosvax.com (M.F.); david@diosvax.com (D.A.W.); rebecca@diosvax.com (R.K.); jlh66@cam.ac.uk (J.L.H.); gwc26@cam.ac.uk (G.W.C.); 4 Institute of Medical Microbiology and Hygiene, University of Regensburg, 93053 Regensburg, Germany; Benedikt.Asbach@klinik.uni-regensburg.de (B.A.); ralf.wagner@klinik.uni-regensburg.de (R.W.); 5 Vector Development and Production Laboratory, St. Jude Children’s Research Hospital, Memphis, TN 38105, USA; 6 Department of Veterinary Medicine, University of Cambridge, Cambridge CB3 0ES, UK; 7 Department of Veterinary Public Health and Preventive Medicine, College of Veterinary Medicine, Federal University of Agriculture Makurdi, Makurdi P.M.B. 2373, Bene State, Nigeria; amehodinya@gmail.com; 8 Department of Veterinary Tropical Diseases, Faculty of Veterinary Science, University of Pretoria, P. Bag X04, Onderstepoort 0110, South Africa; SabetaC@arc.agric.za; 9 OIE Rabies Reference Laboratory, Agricultural Research Council-Onderstepoort Veterinary Research, Onderstepoort 0110, South Africa; 10 Animal and Plant Health Agency (APHA), Department of Virology, Weybridge, Surrey KT15 3NB, UK; ashley.banyard@apha.gov.uk; 11 Institute of Clinical Microbiology and Hygiene, University Hospital Regensburg, 93053 Regensburg, Germany

**Keywords:** influenza, hemagglutinin, pseudotype, vaccine, immunogenicity, monoclonal antibody, neutralization

## Abstract

We developed an influenza hemagglutinin (HA) pseudotype library encompassing Influenza A subtypes HA1-18 and Influenza B subtypes (both lineages) to be employed in influenza pseudotype microneutralization (pMN) assays. The pMN is highly sensitive and specific for detecting virus-specific neutralizing antibodies against influenza viruses and can be used to assess antibody functionality in vitro. Here we show the production of these viral HA pseudotypes and their employment as substitutes for wildtype viruses in influenza neutralization assays. We demonstrate their utility in detecting serum responses to vaccination with the ability to evaluate cross-subtype neutralizing responses elicited by specific vaccinating antigens. Our findings may inform further preclinical studies involving immunization dosing regimens in mice and may help in the creation and selection of better antigens for vaccine design. These HA pseudotypes can be harnessed to meet strategic objectives that contribute to the strengthening of global influenza surveillance, expansion of seasonal influenza prevention and control policies, and strengthening pandemic preparedness and response.

## 1. Introduction

Influenza viruses are segmented, negative sense, single-stranded, enveloped RNA viruses belonging to the Orthomyxoviridae family [1,2,3]. Within this family, there are three types of influenza virus that circulate in humans, influenza A, B, and C [4,5,6]. Only influenza A (IAV) and influenza B (IBV) viruses are endemic in the global human population, rapidly spreading around the world in seasonal epidemics, imposing considerable economic burden and death [7,8]. From its wild bird reservoir, IAV is able to transmit from domestic poultry [9], which is the gateway to infection of mammals, most notably, swine and humans [10]. IBV’s natural reservoir is humans, however there have been reports of infection in seals [11,12,13], alluding to its potential to cause disease in other species.

Influenza A and, to a lesser extent, influenza B can be further classified by structural and genetic differences in the two most abundant glycoproteins expressed on the viral surface—hemagglutinin (HA), which is required for viral entry and fusion [14,15,16], and neuraminidase (NA), which is involved in the release of viral progeny [17]. Currently, 18 distinct antigenic HA (H1-H18) and 11 antigenic NA (N1-N11) subtypes have been de-scribed for IAV [6,7,18]. Based on phylogenetic analysis, IAV HA subtypes are divided into two groups: Group 1—H1, H2, H5, H6, H8, H9, H11, H12, H13, H16, H17, and H18 subtypes, and Group 2—H3, H4, H7, H10, H14, and H15 [17]. IBV is not as diverse and has been divided into two distinct lineages, B/Yamagata-like and B/Victoria-like viruses [19].

Hemagglutinin is a trimeric glycoprotein consisting of a globular head attached to a fibrous stem [16,20,21]. The HA head is highly antigenic and is subject to mutations and reassortment of genetic material over time [10,22,23]. Minor genetic changes such as single point mutations in the HA head are known to give rise to antigenic drift. In contrast, antigenic shift, wherein an influenza A virus strain acquires an HA or NA segment from another subtype of IAV, usually from a zoonotic reservoir, can also occur leading to the emergence of new variants or strains [22,24,25,26,27,28,29]. Antigenic shift is of concern as it may result in the emergence of a completely novel virus to which the human population has no pre-existing immunity and, as such, may have pandemic potential. To date only three HA (H1, H2, and H3) and two NA (N1 and N2) subtypes are known to have caused human pandemics [30,31,32,33]. However, this does not preclude other subtypes from causing a pandemic in the human population in the future. There have been numerous documented cases of human infection with highly pathogenic influenza A viruses (HPAI) H5 and H7, viral subtypes that predominantly cause outbreaks in poultry [34,35,36,37]. Nonetheless, these incidences have not yet resulted in these viruses acquiring the ability to sustain human to human transmission [38,39,40]. Whilst antigenic divergence both within and across HA subtypes exists, the HA stem domain is more conserved and, although not as immunogenic as the head domain [17,22], is increasingly being explored as a candidate for universal influenza vaccines [22,41]. As such, the importance of studying HA structure and function and monitoring antigenic changes within HA is critical to understanding antigenic evolution, defining the most antigenically relevant antigens for annual human vaccination programs [42,43], determining potent universal vaccine targets [44,45], developing vaccines for veterinary use [9,46], and improving influenza diagnosis and therapeutic interventions [47,48,49,50].

Vaccine strain selection for seasonal influenza is carried out via the hemagglutinin inhibition (HI) assay that antigenically characterizes influenza viruses [51,52]. The HI test works by measuring the interaction between the serum antibody and the influenza HA domain of currently circulating IAV and IBV strains and the resulting inhibition of red blood cell agglutination and is currently the measure for seroconversion and protection [43,53,54,55]. To improve current vaccination strategies and to aid the development of a universal influenza vaccine, additional reliable tools are necessary to identify and progress promising candidates targeting both the hemagglutinin head and stem domains [49,56,57,58]. The advent of pseudotyped lentiviral vectors have enabled the study of HA interactions with antibodies, drugs, and host cell receptors with ease [13,59,60,61]. These pseudotypes (PV) undergo abortive replication and do not give rise to replication-competent progeny [62,63]. While it is logistically possible to deal with low pathogenic strains of influenza, studies on strains that are exotic and not widespread in the population are considerably hampered by the availability of BSL facilities and highly trained and qualified personnel required for handling and processing these viruses.

To address these issues, we constructed a comprehensive library of IAV and IBV HA pseudotypes that we tested against available antisera and HA stem-directed monoclonal antibodies, to detect neutralizing responses in sera in mouse vaccine studies to produce optimized seasonal vaccines and candidate pandemic vaccines. This repository of pseudotypes is contributing to the World Health Organization’s global influenza strategy for 2019-2030 of “Prevent, Control and Prepare” [64], with the goal of employing these PV as tools to further vaccine R&D that will contribute to reducing the burden of seasonal influenza, minimizing the risk of zoonotic influenza, and mitigating the impact of pandemic influenza.

## 2. Materials and Methods

### 2.1. Plasmid Production and Transformation

Hemagglutinin genes from Influenza A virus (IAV) subtypes HA1-18 and Influenza B (IBV), presplit, B/Victoria-like, and B/Yamagata-like viruses, were cloned in either pI.18 (in house), phCMV1 (GenScript, Leiden, The Netherlands), or pEVAC plasmids (GeneArt, Regensburg, Germany). pI.18 is a high-copy AmpR pUC-based plasmid that permits robust mammalian gene expression in various cell types via the human cytomegalovirus (hCMV) immediate-early gene promoter and the enhancer hCMV Intron A [65]. phCMV1 is a constitutive mammalian gene expression vector driven by a modified hCMV immediate-early promoter and enhancer/intron together with a Simian Vacuolating virus 40 (SV40) promoter with KanR and NeoR allowing selection of plasmid-positive prokaryotic and eukaryotic cells. pEVAC is also a mammalian expression vector with an hCMV immediate-early promoter/enhancer followed by an intron (HTLV-1-R splice donor and hCMV-IE splice acceptor), a bovine growth hormone (BGH) polyadenylation sequence, and KanR gene. All HA genes were gene-optimized and adapted to human codon use using the GeneOptimizer algorithm [66] and have a strong Kozak-initiation motif.

Influenza hemagglutinin plasmid constructs were generated by cloning the IAV or IBV HA transgenes into pI.18, phCMV1, or pEVAC via restriction digest into the plasmids’ multiple cloning site (MCS). Plasmids were transformed in chemically induced competent *E. coli* DH5α cells (Invitrogen 18265-017) via the heat-shock method. Plasmid DNA was recovered from transformed bacterial cultures via the plasmid mini kit (Qiagen 12125, Manchester, UK) or the endotoxin-free HiSpeed Plasmid Midi Kit (Qiagen 12643, Manchester, UK). All DNA extracts were quantified using UV spectrophotometry (NanoDrop™—Thermo Scientific, Paisley, UK).

### 2.2. Propagation and Maintenance of Cell Cultures

Human embryonic kidney (HEK) 293T/17 (ATCC: CRL-11268ª) cells were used for production and titration of pseudotyped lentiviral vectors and neutralization assays. Madin–Darby canine kidney (MDCK) II cells were used for titration and neutralization assays of Influenza H17 and H18 pseudotyped viruses. Both cell lines were maintained in complete medium, Dulbecco’s modified essential medium (DMEM) (PANBiotech P04-04510, Wimborne, UK) with high glucose and GlutaMAX. DMEM was supplemented with 10% (*v*/*v*) heat-inactivated foetal bovine serum (PANBiotech P30-8500), and 1% (*v*/*v*) penicillin–streptomycin (PenStrep) (Sigma Aldrich, Dorset, UK P4333). Cells were incubated at 37 °C and 5% CO_2_.

### 2.3. Production of Influenza HA Pseudotypes (PV)

Influenza HA pseudotypes were produced as described previously [13,60]. Briefly, 4 × 10^5^ HEK 293T/17 cells in complete DMEM were seeded per well of a 6-well plate and incubated at 37 °C, 5% CO_2_ overnight. The next day, media was replaced and cells were transfected using Opti-MEM™ (Thermo Fisher Scientific 1985062, Paisley, UK) and FuGENE^®^ HD transfection reagent (Promega E2312 Madison, USA) with the following plasmids: HA encoding plasmid (pI.18/phCMV1/pEVAC), luciferase reporter plasmid pCSFLW [59], and p8.91 gag-pol (Gag-Pol expression plasmid [62,63,67]). Plates were incubated at 37 °C, 5% CO_2_. For transfection of low pathogenicity avian influenza (LPAI) and other subtypes with a monobasic cleavage site, an additional plasmid expressing type II transmembrane protease serine 2 (TMPRSS2) [68], type II transmembrane protease serine 4 (TMPRSS4) [69], or human airway trypsin-like protease (HAT) [68] was also included. For the H18 subtype, 50 ng of A/flat-faced bat/Peru/033/2010/N11 in pEVAC was also included. The amounts of plasmid DNA and reagents used for transfection in a single well of a 6-well plate are indicated in Table 1. All plasmid DNA were combined in Opti-MEM and FuGENE^®^ HD added dropwise followed by incubation for 15 min. The plasmid DNA-OptiMEM mixture was then added to the cells with constant swirling. At least 8 h post-transfection, 1 unit of exogenous neuraminidase (Sigma AldrichN2876, Paisley, UK) was added to the 6 well-plates, with the exception of the H18 subtype. Forty-eight hours post-transfection, supernatants were collected, passed through a 0.45 μm filter, and stored at −80 °C.

### 2.4. Influenza Pseudotype Titration

Titration experiments were performed in Nunc F96 MicroWell white opaque polystyrene plates (Thermo Fisher Scientific 136101). The pseudotype production titre was evaluated by transducing HEK293T/17 cells (or MDCKII cells for H17 and H18) with the PV. Fifty microliters of viral supernatant were serially diluted two-fold across a 96-well plate in duplicate before adding 50 μL of 1 × 10^4^ HEK293T/17 cells to each well. Control wells in which there was no PV added were also present on each plate as an indirect cell viability measurement. Plates were then incubated at 37 °C, 5% CO_2_ for 48 h. Media was removed and 25 µL of Bright-Glo^®^ (Promega, Madison, USA) luciferase assay substrate was added to each well. Titration plates were then read using the GloMax^®^ Navigator (Promega, Southampton, UK) using the Promega GloMax^®^ Luminescence Quick-Read protocol. Viral pseudotype titre was then determined in relative luminescence units/mL (RLU/mL).

### 2.5. Reference Antisera and Bat Surveillence Sera

Reference antisera to assess the neutralization sensitivity of representative IAV and IBV pseudotypes from our library were obtained from the OIE (World Organisation for Animal Health), the National Institute for Biological Standards and Control (NIBSC), or the Animal and Plant Health Agency (APHA) (Table 2). Antisera were generated by immunizing chickens (OIE) and sheep (NIBSC) with HA antigen. At the time of publication, reference antisera for H17 and H18 were not available, however, frugivorous bat sera, collected as part of a bat sera surveillance program in Nigeria, was provided by APHA.

### 2.6. Mouse Immunogenicity Studies

**Figure 1:**
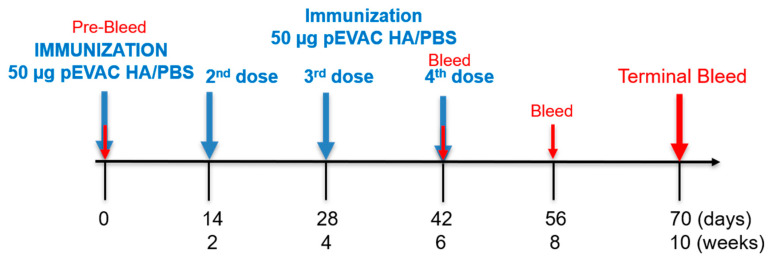
Study schedule of immunization with pEVAC HA antigens. Mice received either pEVAC HA antigens or PBS (negative control groups) on weeks 0, 2, 4, and 6 via subcutaneous rear flank injection. Blood was collected on weeks 6, 8, and 10.

For mouse immunogenicity studies, 6–8-week-old female BALB/c mice were obtained from Charles River Laboratories and housed at the University Biomedical Services, University of Cambridge. Mice were divided into groups of six for each individual vaccination antigen. On day 0, mice were injected subcutaneously (SC) on the rear flank with a 50 μL volume of 50 µg of pEVAC HA, produced using the EndoFree Plasmid Mega Kit (Qiagen Manchester, UK)), or negative control group (PBS) for negative control groups. Immunizations were repeated on weeks 2, 4, and, 6 (Figure 1). Mice were weighed daily and monitored for any signs of disease or distress. Mice were bled at 42 days post immunization (dpi), 56 dpi, and 70 dpi (Figure 1). At 70 days post immunization, all mice were culled and terminal bleeds collected. Collected blood was left to clot for 1 h at room temperature and serum was separated via centrifugation at 2000× *g* for 10 min at 4 °C and stored at −20 °C.

### 2.7. Pseudotype Microneutralization (pMN) Assay

We performed pseudotype microneutralization assays using standard reference antisera, monoclonal antibodies (mAb), and serum samples from animal studies. The monoclonal antibody concentrations used were in the range of 0.5–1000 ng/mL and serum and antiserum samples were initially diluted 1:20 or 1:50 in 50 µL of complete DMEM, before being serially diluted two-folds across a 96-well plate. Fifty microliters of PV at a titre of 1.0 × 10^6^ RLU/well as determined via titration was then added to the mAb or serum dilutions, making the final dilution of sera 1:40 or 1:100. This mixture was incubated for 1 h at 37 °C, 5% CO_2_. Afterwards, 50 µL of 1.5 × 10^4^ HEK293T/17 cells were added to each well. PV only (equivalent to 0% neutralization) and cell only controls with no virus (equivalent to 100% neutralization control) were also included in the test plate. Plates were incubated for 48 h at 37 °C and 5% CO_2_. Media was removed and 25 µL of the Bright-Glo^®^ luciferase assay substrate added to each well. Plates were then read using the GloMax^®^ Navigator (Promega, Southampton, UK) using the Promega GloMax^®^ Luminescence Quick-Read protocol. Half-maximal inhibitory dilution or concentration (IC_50_) values were calculated using GraphPad Prism 8.12. A detailed analysis is described in Ferrara, 2018 [70].

### 2.8. Statistical Analysis

All statistical analyses were performed with GraphPad Prism 8.12 for Windows (GraphPad Software, San Diego, CA, USA). The Kruskal–Wallis H test, a rank-based nonparametric test, was used to determine if there were statistically significant differences between two or more groups in comparison to a control group.

### 2.9. Bioinformatic Analysis

HA sequences for both IAV and IBV were downloaded from the Influenza Virus Resource database (IVRD) (fludb.org). The phylogenetic tree was generated using the Cyber-Infrastructure for Phylogenetic Research (CIPRES) gateway [71]. The resulting tree file was then visualized using the Archaeopteryx tree viewer in the Influenza Resource Database (IRD) [72].

## 3. Results

### 3.1. Production of the IAV and IBV Pseudotype Library

**Figure 2:**
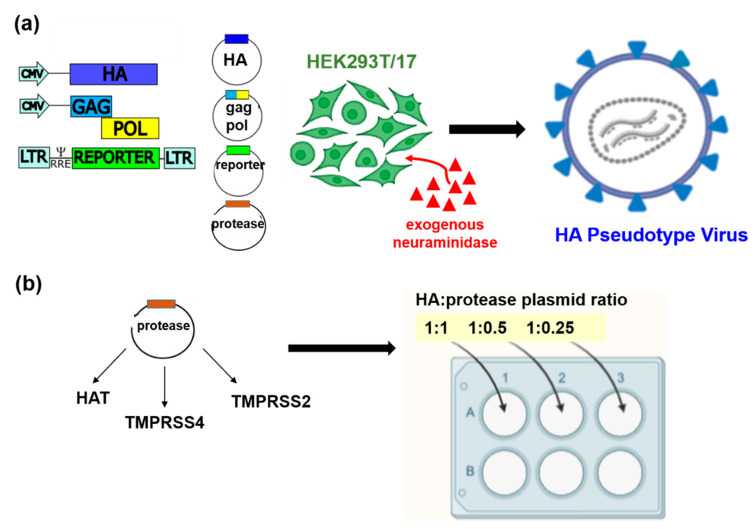
Schematic representation of the production of influenza HA pseudotypes by plasmid transfection. (**a**) Using the 3 or 4 plasmid system, exogenous NA was added to the transfected cultures at least 8 h post-transfection. (**b**) Addition of protease, which is necessary for the production of low pathogenic influenza A viruses (LPAI) and subtypes with a monobasic cleavage site, and IBV were optimized to increase titres by transfecting in a ‘checkerboard’ approach with different proteases (e.g., HAT, TMPRSS4, and TMPRSS2). Protease plasmid was added at a ratio of 1:1, 1:0.5, and 1:0.25 to HA plasmid DNA for rapid optimization in a 6 well plate format. All pseudotypes were harvested after 48 h in culture. The image was created using BioRender (biorender.com).

The influenza pseudotype viruses (PV) described herein were constructed using the transfection method detailed above (Section 2.3). All PV were produced with the following three plasmids: (i) a plasmid containing packaging genes from a surrogate lentivirus (HIV) (gag-pol), which is defective for the native HIV envelope, (ii) a plasmid expressing the HA envelope of the strain being studied (IAV or IBV), and (iii) a transfer plasmid expressing the firefly luciferase reporter (Figure 2a). One unit of exogenous neuraminidase (exoNA) was added per well to facilitate viral egress, with the PV containing the HA envelope on its surface, harvested in cell supernatants. For influenza H18, an additional plasmid expressing A/flat-faced bat/Peru/033/2010/N11 was included in the place of exogenous NA

**Figure 3:**
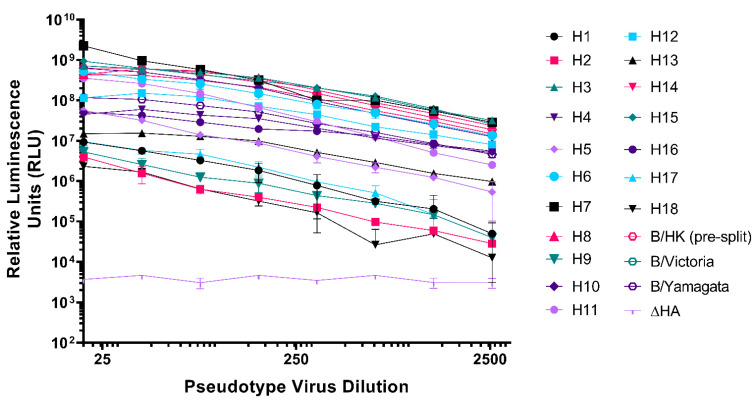
Titration of representative Influenza A (H1-H18) and influenza B (presplit, B/Victoria-like and B/Yamagata-like lineages) viruses. Pseudotyped lentiviral particles with HA envelopes: H1—A/England/195/2009(H1), H2—A/quail/Rhode Island/16-0186222-1/2016(H2), H3—A/ruddy turnstone/Delaware Bay/606/2017(H3), H4—A/green-winged teal/California/K218/2005(H4), H5—A/gyrfalcon/Washington/41088-6/2014(H5), H6—A/American wigeon/California/HS007A/2015(H6), H7—A/Shanghai/2/2013(H7), H8—A/mallard/Netherlands/7/2015(H8), H9—A/chicken/Israel/291417/2017(H9), H10—A/duck/Bangladesh/24268/2015(H10), H11—A/red shoveler/Chile/C14653/2016(H11), H12—A/northern shoveler/Nevada/D1516557/2015(H12), H13—A/laughing gull/New Jersey/UGAI7-2843/2017(H13), H14—A/mallard/Astrakhan/263/1982(H14), H15—A/duck/Bangladesh/24697/2015(H15), H16—A/black-headed gull/Netherlands/1/2016(H16), presplit—B/Hong Kong/8/1973, B/Victoria—B/Brisbane/60/2008, and B/Yamagata—B/Phuket/3073/2013, were titrated in HEK293T/17 cells. H17—A/little yellow-shouldered bat/Guatemala/60/2017(H17), and H18—A/flat-faced bat/Peru/33/2010(H18) were titrated in MDCKII cells. ΔHA is included as a no envelope control. Each point represents the mean and standard deviation of two replicates per dilution (*n* = 2). Readout is expressed in relative luminescence units (RLU).

IAV and IBV strains that contain monobasic cleavage sites require the presence of a trypsin-like protease in vitro to catalyse HA proteolytic cleavage from the inactive trimeric HA0 to the active HA1 and HA2 leading to viral membrane fusion [73,74,75]. As demonstrated previously, an additional plasmid expressing a trypsin-like protease was required for PV production (Figure 2a) [60,68,69], with the amount of protease plasmid DNA requiring optimization for each PV produced. We found that this is dependent on the HA subtype and occasionally the strain being produced (Figure 3, Table 3). Optimization was achieved using a 6-well plate checkerboard system for protease amounts (Figure 2b), and a fixed amount of all other plasmids was used to transfect 293T/17 cells. For all strains except HPAI strains, initial transfections were undertaken with human airway trypsin-like protease (HAT) in the top three wells and transmembrane serine protease 4 (TMPRSS 4) in the bottom three wells of the 6-well plate. Protease plasmid was added at a ratio to the HA plasmid (Figure 2b), i.e., for every 10 ng of HA, we tested with 10 ng, 5 ng, and 2.5 ng protease DNA. All PV produced were titrated and the PV titre determined in RLU (Figure 3).

If production titres were less than 5 × 10^7^ RLU/mL, we additionally tested with transmembrane serine protease 2 (TMPRSS2) for the PV strain in the same plasmid ratios (Figure 2b). Generally, TMPRSS4 produced the highest titres (RLU/mL) for all subtypes except for H17 and IBV lineages, where HAT produced the highest titres and H2, H3, and H4 required TMPRSS2 [76] for optimal production (Table 3). Protease was not necessary for the production of HPAI representative viruses, H5 and H7 in Figure 3 and Table 2 (as indicated by *). Optimized conditions were then recorded and PV production was scaled up to produce larger PV stocks. We showed here (Figure 3, Table 3) the highest titres we achieved during several rounds of PV production following optimization. PV titre variability between batches was found to be small, with titres achieved within half a log of each other.

**Figure 4:**
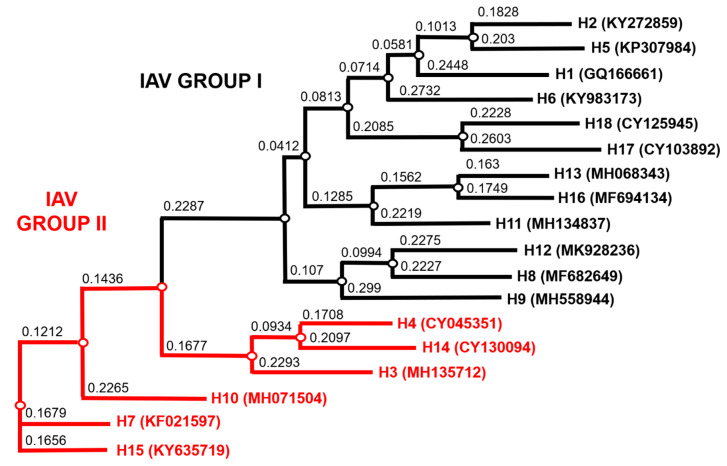
Phylogenetic tree of representative IAV HA from the PV library constructed as shown in Figure 3 and Table 3. Influenza A Group I HA PV are shown in black and IAV Group II PV in red. Accession numbers are reported with the subtype on the tree tips. Nodes are shown at the ends of branches, which represent sequences or hypothetical sequences at various points in evolutionary history. Branch lengths indicate the extent of genetic change. The tree generated was constructed with PhyML on the Influenza Research Database (IRD) [72] and graphically elaborated with Archaeopteryx.js (https://sites.google.com/site/cmzmasek/home/software/archaeopteryx-js) (access on 3 April 2021).

Our optimized method enabled us to produce the most comprehensive pseudotype library to date with representative strains from IAV subtypes H1-H18 and both IBV lineages. Figure 4 illustrates the range of IAV subtypes already present and available in this library. Full details of the current library at the VPU are indicated in Table S1. These include low pathogenic avian influenza (LPAI) strains from H7, in addition to HPAI H5 and H7 presented (Figure 3, Table 3).

### 3.2. Neutralization of Pseudotypes by Reference Antisera

**Figure 5:**
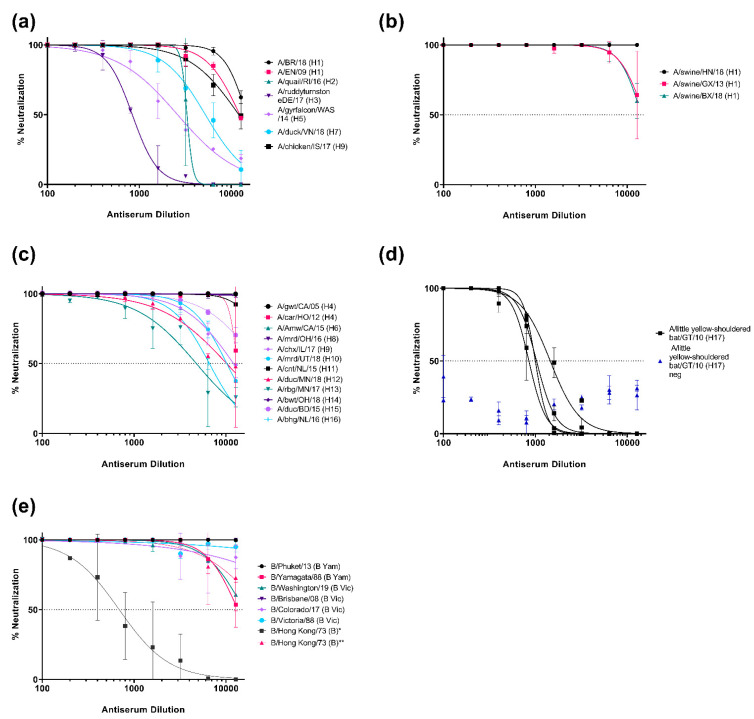
Neutralization of influenza pseudotypes by reference antisera and bat sera from influenza surveillance. (**a**) Neutralization of representative IAV subtypes that have previously caused infection in humans (H1, H2, H3, H5, H7, and H9). (**b**) Neutralization of pseudotypes representing IAV isolated from swine (H1). (**c**) Neutralization of pseudotypes that are representative of IAV found in avian populations (H4, H6, H8, H9, H10, H11, H12, H13, H14, H15, and H16). (**d**) Neutralization of H17 PV (A/little yellow-shouldered bat/Guatemala/060/2010) by bat sera from bat surveillance sampling in Nigeria as provided by APHA. (**e**) Neutralization of IBV pseudotypes that have caused human infection (B/Yamagata-like and B/Victoria-like viruses and presplit IBV). As presplit antiserum was not available, neutralization susceptibility of this PV to B/Yamagata lineage antisera (denoted with *) and B/Victoria lineage antisera denoted with **) have been shown. Neutralization was measured by a luciferase reporter assay. Reference antisera and bat sera were serially diluted two-fold from a starting dilution of 1:100. A total of 1.0 × 10^6^ RLU of PV was then added to each well. For all plots, each point represents the mean and standard deviation of two replicates per dilution. Details of reference antisera are indicated in Table 2.

The neutralization susceptibility of representative PV generated to the available HA subtype specific reference antisera (Table 2) was assessed. All reference antisera were able to neutralize the subtype homologous PV they were tested against (Figure 5). We showed neutralization dose response curves for PV representing IAV strains, which were reported as the cause of human disease, including avian subtypes that caused zoonotic infection without being associated with sustained human to human transmission (HPAI H5 and H7, and H9), with IC_50_ dilution values ranging from the most potent, ~14521 for H1, and the least potent, ~416 for H3 (Figure 5a). We also tested against HA PV that have been associated with swine and human infection, such as H1 strains that have been found in pigs, which may acquire the ability to transmit to humans due to possible antigenic shift (Figure 5b), and avian IAV subtypes that are found in their natural reservoir, wild, and occasionally domesticated, birds (Figure 5c) and may evolve in the future to cause novel pandemic strains in humans. Antisera used herein have IC_50_ dilution values that are comparatively higher than the IC_50_ values for HA subtypes associated with human disease (ranging from ~13,000 for H14 to ~900 for H13). Currently there is no commercially available H17 or H18 subtype antisera. Due to the association of H17 in frugivorous bat species, sera collected from bats in Nigeria, as provided by the Animal and Plant Health Agency (APHA), were assessed against the H17 pseudotype (Figure 5d). Three bats within a larger panel (only five samples shown here) neutralized the H17 PV with similar IC_50_ dilution values of 537, 645, and 724, respectively. (Figure 5d). Human IBV PV from both Yamagata and Victoria-like lineages were also susceptible to neutralization from reference antisera (Figure 5e). Antisera to pre-split IBV strains were not available for this study, nonetheless, our representative pre-split IBV (B/Hong Kong/73) strain was neutralized by both anti-Yamagata and anti-Victoria reference sera. However, this PV was more susceptible to neutralization by antisera generated using HA from the Victoria-like lineage than the Yamagata-like lineage (Figure 5e), with IC_50_ dilution values of 688.8 and >10,000, respectively. All antisera IC_50_ dilutions are reported in Table S2.

### 3.3. Mouse Immunogenicity Studies

We conducted preliminary mouse immunogenicity studies to determine the capacity of selected HA potential vaccine candidates to elicit a measurable immune response, assess safety, and to see if our dose and dosing regimen could inform future preclinical trials for protection and efficacy. In this study, we measured immune responses to vaccination in mouse sera (humoral immune responses). The humoral immune response is assessed from the post-vaccination appearance of antibody directed at the specific vaccine antigen at appointed time points. Using our PV library, we measured functional antibodies in mouse sera that can be applied to samples in low containment, which would not be possible using wildtype viruses.

#### 3.3.1. Monitoring of the Immune Response throughout the Vaccination Protocol

We first determined if our dose of 50 µg of pEVAC HA DNA could generate an immune response that could be monitored across a certain time frame, and if additional immunizations could increase this specific immune response (Figure 1). It should be noted that all mice used for our experiments were naïve and have not had prior exposure to influenza, hence it is assumed that neutralization of PV will be due to the immune response generated by immunization with influenza antigens.

**Figure 6:**
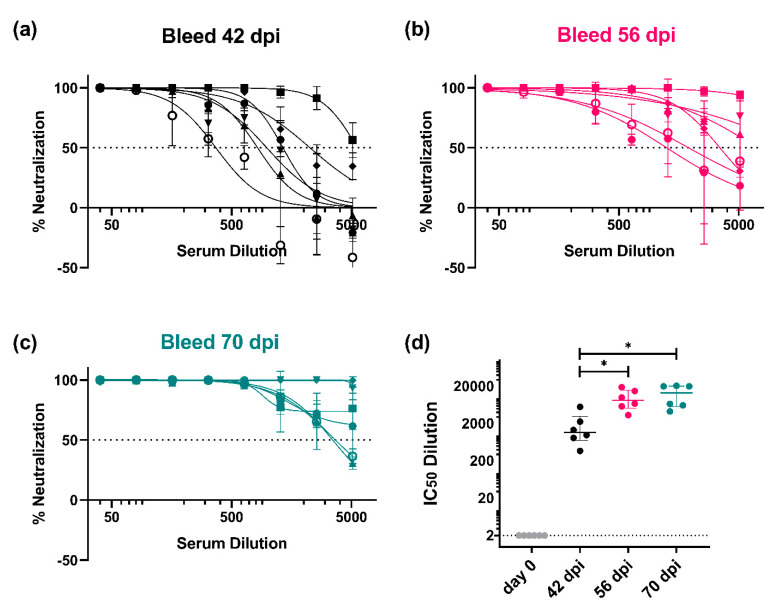
In vitro neutralizing activity of mouse sera against A/England/195/09 (H1) (EN/09) as monitored at specific timepoints during the immunization protocol. Mice were vaccinated with 50 µg of pEVAC EN/09 (H1) on days 0, 14, 28, and 42 (Figure 1). Bleeds were taken (**a**) 42 days post immunization (dpi), (**b**) 56 dpi, and (**c**) 70 dpi (terminal bleed). Neutralizing activity was tested against 1 × 10^6^ RLU of A/England/195/09 (H1) PV. (**d**) Comparison of the half maximal inhibitory dilutions (IC_50_) in post-vaccination samples as a function of time is shown in brackets (* *p* < 0.05). The broken line shows an assigned baseline level of 2 indicating 0% neutralization. For plots (**a**–**c**), the mean and standard deviation of individual mouse serum samples are shown (*n* = 6). Plot (**d**) shows the median and interquartile range of samples tested.

We observed an immune response in all mice (*n* = 6) vaccinated with pEVAC EN/09 (H1) against the corresponding homologous EN/09 (H1) PV in all post-vaccination samples from the earliest time of sampling (42 dpi) as compared to pre-vaccination sera (day 0) (Figure 6). At 42 dpi, mice had received three immunizations (day 0, day 14, and day 28) and had already developed neutralizing antibody responses compared to day 0 (Figure 6a). At 56 and 70 dpi, mice had received four immunizations (day 0, day 14, day 28, and day 42) (Figure 6b,c). A significant increase in detectable neutralizing antibodies can be seen between the third and fourth round of immunizations (Figure 6d) demonstrating that boosting the immune response with subsequent immunizations may give rise to stronger neutralizing titres. There was no significant difference between neutralizing activity of mouse sera from bleeds taken at 56 and 70 dpi (Figure 6d), suggesting that we had employed an ideal number and interval of immunizations of pEVAC HA to achieve optimal strain-specific titres in mice.

#### 3.3.2. Strain-Specific and Subtype-Specific Immune Responses Post-Vaccination

The ability of mouse sera vaccinated with a specific IAV strain to neutralize strains of the same subtype was then evaluated. This was to assess possible strain cross-reactivity of the immune responses elicited by vaccination. This is especially important for influenza, which is subject to continuous random antigenic drift and wherein viruses of the same subtype may belong to different clades. Given this, we chose to investigate H1, the most recent IAV pandemic strain (in 2009), and HPAI subtypes H5 and H7, which have caused human spillovers from fatal poultry outbreaks in the past. We employed the same immunization procedure as detailed above and terminal bleeds were assessed for neutralizing activity.

**Figure 7:**
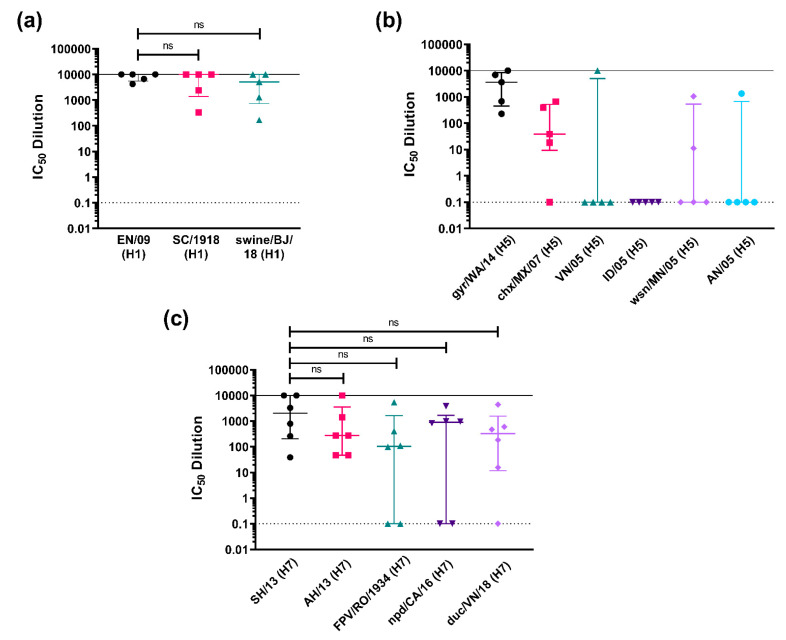
In vitro neutralizing activity as shown by IC_50_ dilution of mouse sera vaccinated with an HA subtype tested against homologous PV and representative PV strains of the same subtype. (**a**) Mice were vaccinated with 50 µg of pEVAC HA A/England/195/09 (H1) (EN/09) (*n* = 5). Terminal bleeds (70 dpi) were tested against H1 PV strains (x-axis): homologous EN/09, A/South Carolina/1/1918 (H1) (SC/1918), and A/swine/Beijing/301/18 (H1) (swine/BJ/18). (**b**) Mice were vaccinated with 50 µg of pEVAC HA A/gyrfalcon/Washington/41088-6/14 (H5) (gyr/WA/14) (*n* = 5). Terminal bleeds (70 dpi) were tested against H5 PV strains (x-axis): homologous gyr/WA/14, A/chicken/Mexico/7/07 (H5) (chx/MX/07), A/Indonesia/5/05 (H5) (ID/05), A/Vietnam/1203/04 (H5) (VN/05), A/whooper swan/Mongolia/244/05 (H5) (wsn/MN/05), and A/Anhui/1/05 (H5) (AN/05). (**c**) Mice were vaccinated with 50 µg of pEVAC HA A/Shanghai/2/13 (H7) (SH/13) (*n* = 6). Terminal bleeds (70 dpi) were tested against H7 PV strains (x-axis): homologous SH/13, A/Anhui/1/13 (H7) (AH/13), A/FPV/Rostock/1934 (H7) (FPV/RO/1934), A/northern pintail duck/California/UCD1582/16 (H7) (npd/CA/16), and A/duck/Vietnam/HU10-64/18 (H7) (duc/VN/18). For all plots, the median and interquartile range of individual mouse serum samples per immunization group are shown. The solid line indicates an assigned maximum IC_50_ dilution of 10,000 showing 100% neutralization and the broken line shows an assigned baseline level of 0.1 indicating 0% neutralization (cell only mean). Comparisons of no significant difference (ns: *p* > 0.05) against the homologous PV are shown in brackets.

For our A/H1 panel, we immunized mice with the pandemic strain EN/09 (H1) (Figure 7a). We tested against a previous H1 pandemic strain, SC/1918 (Spanish flu), and a possible emerging pandemic strain, swine/BJ/18, guided by the knowledge that the last H1N1 pandemic (swine flu) was caused by a quadruple-reassortant virus, containing genes from Asian and European swine, North American avian, and human influenza virus [32]. Terminal sera from mice immunized with EN/09 were able to neutralize all H1 PV tested, with no significant difference observed in neutralization activity against homologous and heterologous strains of the same subtype, with representative PV strains covering 100 years, from 1918 to 2018 (Figure 7a).

We immunized mice with gyr/WA/15 (H5) for our A/H5 panel (Figure 7b). We tested across six different clades, gyr/WA/14 (clade 2.3.4.4c), chx/MX/07 (American non-goose Guangdong), ID/05 (clade 2.1.3.2), VN/04 (clade 1), wsn/MN/05 (clade 2.2), and AN/05 (clade 2.3.4). HPAI H5 strains have been known to cause deadly outbreaks in poultry with some human spillover in the past [77,78]. H5 viruses especially those in clade 2 are known to evolve rapidly and extensively, with newly emerging strains circulating in many regions of the world [78]. Our findings here demonstrate that terminal sera from mice immunized with gyr/WA/15 (H5) were unable to neutralize the other H5 PV tested as effectively as the homologous strain used for vaccination (Figure 7b). Interestingly, one mouse developed a broadly neutralizing response and was able to neutralize all PV tested except for IN/05 but all other samples revealed no H5 cross-strain neutralizing immune response (Figure 7b).

Mice were immunized with SH/13 (H7) for the H7 panel (Figure 7c). In addition to the homologous SH/13 (H7) PV, we tested against four other H7 strains, FPV/RO/1934, the historical H7 fowl plague virus of 1934, a human IAV PV, AH/13, and two avian PV, npd/CA/16, and duc/VN/18. Terminal sera from mice immunized with SH/13 were able to neutralize all H7 PV tested, with no significant difference observed in the means of the IC_50_ dilution values obtained against homologous and heterologous strains of the same subtype (Figure 7c). Some serum samples were unable to neutralize all three H7 avian PV, but all serum terminal bleeds were effective against the other H7 human PV tested, AH/13, with neutralization of the homologous strain showing the same pattern (Figure 7c).

We then examined the breadth of responses within a subtype with the idea that one vaccination could protect from small changes caused by antigenic drift and provide some initial protection from reassortant viruses that can transmit between species. This is also the basis of strain selection for seasonal influenza vaccination [56,79]. To test this, we examined cross strain neutralization in mice vaccinated with antigens from strains of IAV H3 isolated from human and avian origins. H3 circulates in the human population and is a component of the quadrivalent influenza vaccine, transmission is often from animal sources [7,8], and therefore cross-reactive immune responses would be beneficial.

**Figure 8:**
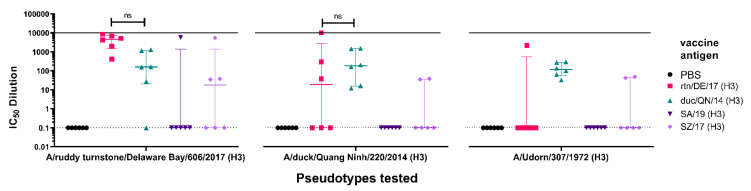
In vitro neutralizing activity as shown by IC_50_ dilution of mouse sera vaccinated with avian and human pEVAC H3 vaccine antigens tested against homologous avian H3 PV and a representative human PV strain of H3. Four groups consisting of 6 mice each (*n* = 6/group) were vaccinated with 50 µg of pEVAC HA-A/ruddy turn-stone/Delaware Bay/606/2017 (H3) (rtn/DE/17) (pink square) or HA-A/duck/Quang Ninh/220/2014 (H3) (duc/QN/14) (green triangle), HA-A/South Australia/34/2019 (H3) (SA/19) (violet inverted triangle), and HA-A/Switzerland/8060/2017 (H3) (SZ/17) (purple diamond), respectively. An additional group of mice was vaccinated with PBS (negative control group) (*n* = 6). Terminal bleeds (70 dpi) were tested against H3 PV strains: 2 homologous avian PV, rtn/DE/17 and duc/QN/14, and one human PV, A/Udorn/307/1972 (UD/1972), as shown in the x-axes. For all plots, the median and interquartile range of individual mouse serum samples per immunization group are shown. The solid line indicates an assigned maximum IC_50_ dilution of 10,000 showing 100% neutralization and the broken line shows an assigned baseline level of 0.1 indicating 0% neutralization (cell only mean). Comparisons of no significant difference (ns: *p* > 0.05) against the homologous PV are shown in brackets.

We immunized groups of mice with H3 from avian strains, A/ruddy turnstone/ Delaware Bay/606/2017 (H3) (rtn/DE/17) and A/duck/Quang Ninh/220/2014 (H3) (duc/QN/14), and two strains of H3 of clades 3C.2a2 that have circulated recently in the human population, A/South Australia/34/2019 (H3) (SA/19) and A/Switzerland/8060/2017 (H3) (SZ/17), respectively (Figure 8). Terminal sera from mice were tested against two avian H3 PV matched to the immunization antigens, rtn/DE/17 and duc/QN/14, and one representative human strain PV, A/Udorn/307/1972 (H3).

Terminal sera from mice immunized with rtn/DE/17 (Figure 8, 1st panel) were able to strongly neutralize a homologous PV (rtn/DE/17) with an IC_50_ dilution range of 413–8634. These mice were also able to neutralize a heterologous PV duc/QN/14 with no significant difference compared to sera from mice vaccinated with the duc/QN/14 antigen (Figure 8, 2nd panel). Only one mouse from the group vaccinated with rtn/DE/17 produced responses that were able to neutralize human H3 PV, A/Udorn/307/1972 (Figure 8, 3rd panel), whereas, all mice vaccinated with duc/QN/14 were able to neutralize A/Udorn/307/1972. This is promising as vaccination with an avian H3 has shown neutralization of a human H3 PV, albeit an older strain from 1972. Mice immunized with SA/19, a human H3 antigen, did not neutralize any of the PV tested except for serum from one mouse, which was able to neutralize PV rtn/DE/17 (IC_50_ dilution of 5659). Sera from half of the mice immunized with SZ/17 were able to neutralize PV rtn/DE/17 and two mice were able to neutralize duc/QN/14 and A/Udorn/307/1972 PV. Our results show that vaccination with duc/QN/14 elicited the best immune responses against all H3 PV tested, either avian or human (Figure 8).

#### 3.3.3. Cross-Subtype Immune Responses Post-Vaccination

As seen with the results indicated above (Figure 7 and Figure 8), vaccination with subtype specific antigens had very little effect against other strains from that same subtype. This is what is observed in seasonal vaccination that generates subtype-specific antibodies that will have little or no efficacy against drifted strains [80,81]. An immunization that gives rise to broadly protective humoral immunity against influenza remains a sought-after goal [82]. It has been previously described that cross-protective immunity against pandemic H1N1 (2009) can be induced by seasonal influenza A (H3N2) infection [83]. Here we attempted to demonstrate cross-subtype neutralization from immunization among IAV subtypes that are closest to each other on the phylogenetic tree for IAV (Figure 4) and between the two IBV lineages, B/Victoria (B/Vic) and B/Yamagata-like viruses (B/Yam).

**Figure 9:**
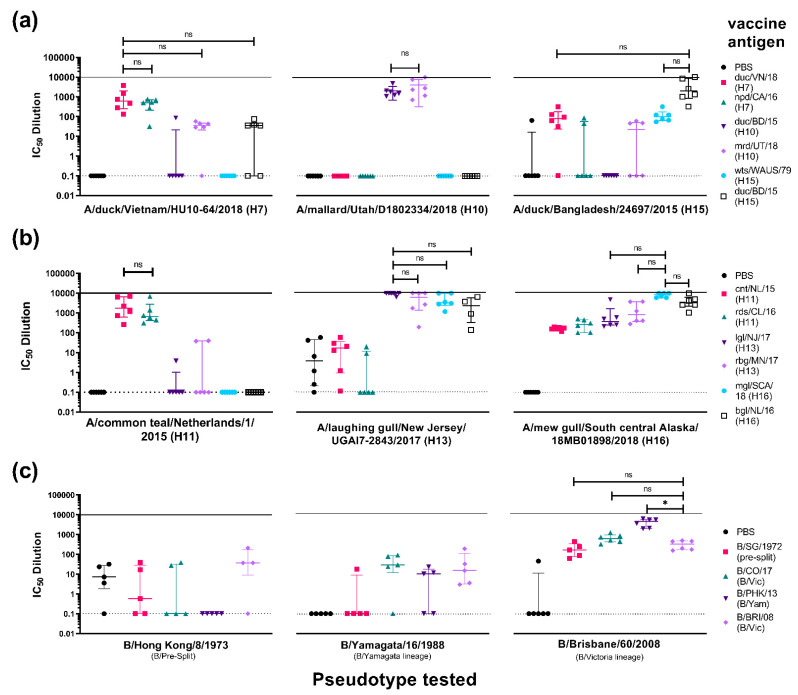
In vitro neutralizing activity as shown by IC_50_ dilution of mouse sera vaccinated with HA antigens from the closest phylogenetically related IAV subtypes, (**a**) H7, H10, and H15, (**b**) H11, H13 and H16, and (**c**) IBV HA antigens from pre-split, Yamagata and Victoria like-lineages. Neutralizing activity of sera from vaccinated mice were tested against homologous and heterologous strains from the same subtype and a representative strain within the related subtypes. (**a**) IAV H7/H10/H15 study. Six groups consisting of 6 mice each (*n* = 6/group) were vaccinated with 50 µg of pEVAC expressing A/duck/Vietnam/HU10-64/2018 (H7) (duc/VN/18) (pink square), A/northern pintail duck/California/UCD1582/2016 (H7) (npd/CA/16) (green triangle) (*n* = 6), A/duck/Bangladesh/24268/2015 (H10) (duc/BD/15) (violet inverted triangle), A/mallard/Utah/D1802334/2018 (H10) (mrd/UT/18) (purple diamond), A/wedge-tailed shearwater/Western Australia/2576/1979 (H15) (wts/WAUS/79) (light blue circle), and A/duck/Bangladesh/24697/2015 (H15) (duc/BD/15) (hollow black square), respectively. Terminal bleeds (70 dpi) were tested against H7 PV duc/VN/18, H10 PV mrd/UT/18, and H15 PV duc/BD/15 as shown on the x-axes. (**b**) IAV H11/H13/H16 study. Six groups consisting of 6 mice each (*n* = 6/group) were vaccinated with 50 µg of pEVAC cloned with A/common teal/Netherlands/1/2015 (H11) (cnt/NL/15) (pink square), A/red/shoveler/Chile/C14653/2016 (H11) (rds/CL/16) (green triangle), A/laughing gull/New Jersey/UGAI7-2843/2017 (H13) (lgl/NJ/17) (violet inverted triangle), A/ring-billed gull/Minnesota/OPMNAI0816/2017 (H13) (rbg /MN/17) (purple diamond), A/black-headed gull/Netherlands/1/2016 (H16) (bhg/NL/16) (light blue circle), and A/mew gull/South central Alas-ka/18MB01898/2018 (H16) (mgl/SCA/18) (black hollow square). Terminal bleeds (70 dpi) were tested against H11 PV cnt/NL/15, H13 PV lgl/NJ/17, and H16 PV mgl/SCA/18 as shown in the x-axes. (**c**) IBV cross liA neage study. Four groups of mice were vaccinated with 50 µg of pEVAC cloned with B/Singapore/222/1979 (presplit) (B/SG/1979) (pink square) (*n* = 5), B/Colorado/06/2017 (B/Vic) (B/CO/17) (green triangle) (*n* = 6), B/Phuket/3073/2013 (B/Yam) (B/PHK/13) (violet inverted triangle) (*n* = 6), and B/Brisbane/60/2008 (B/Vic) (B/BRI/08) (purple diamond) (*n* = 6), respectively. Terminal bleeds (70 dpi) were tested against a representative B pre-split PV, B/Hong Kong/8/1973, a representative B/Yamagata PV, B/Yamagata/16/1988, and B/Victoria PV B/Brisbane/60/2008 as shown in the x-axes. For all plots, an additional group of mice was vaccinated with PBS (*n*=5/6). Plots show the median and interquartile range of individual mouse serum samples per immunization group. The solid line indicates an assigned maximum IC_50_ dilution of 10,000 showing 100% neutralization and the broken line shows an assigned baseline level of 0.1 indicating 0% neutralization. Comparisons of no significant difference (ns: *p* > 0.05) and significant difference (* *p* < 0.05) among IC_50_ dilution values with antigen homologous to the PV being tested against is shown in brackets. In the case of (**c**), comparison is made with neutralization of the antigen belonging to the same lineage as the PV it is tested against.

For the IAV H7/H10/H15 study (Figure 9a, 1st panel), mice vaccinated with npd/CA/16 (H7) showed no significant difference in neutralizing activity (n.s.) with mice vaccinated with duc/VN/18 (H7) when tested against the duc/VN/18 (H7) PV. This was also observed with mice vaccinated with mrd/UT/18 (H10) and duc/BD/15 (H15) (Figure 9a, 1st panel). This suggests that the vaccination with northern pintail duck/CA/16 (H7), mrd/UT/18 (H10), and duc/BD/15 (H15) produced a similar neutralizing response to the homologous antigen against the duc/VN/18 (H7) PV in vitro. Mice vaccinated with the other antigens, duc/BD/15 (H10) and wts/WAUS/79 (H15), showed little to no neutralization of the H7 PV, this may be partly due to this H15 virus being isolated in 1979, suggesting that this avian H15 diverged between 1979 and 2018 (Figure 9a, 1st panel).

Results for groups tested against mrd/UT/18 (H10) PV are more clear-cut (Figure 9a, 2nd panel), with only groups vaccinated with H10 antigens showing neutralizing activity against the PV. There was also no significant difference between the IC_50_ values against the mrd/UT/18 (H10) PV in the group vaccinated with the other H10, duc/BD/15, to that vaccinated with the homologous mrd/UT/18 (H10) (Figure 9a, 2nd panel). Here, only neutralization of PV by mice vaccinated with the same subtype is demonstrated. Looking at the phylogenetic tree (Figure 4), H10 resides on a different branch than H7 and H15, and therefore it was highly unlikely that cross-subtype neutralization would be observed.

For groups tested against duc/BD/15 (H15) PV, vaccination with the homologous antigen, the other H15 antigen, wts/WAUS/79, and duck/VN/18 (H7) showed neutralizing activity (Figure 9a, 3rd panel). Neutralizing activity of mice vaccinated with all other antigens tested was closer to that of the negative control group (PBS), although a few responders, located above the upper extreme quartile, were observed.

For the IAV H11/H13/H16 study (Figure 9b, 1st panel), mice vaccinated with rds/CL/16 (H11) showed no significant difference in neutralizing activity (n.s.) with mice vaccinated with cnt/NL/15 (H11) when tested against the cnt/NL/15 (H11) PV. This suggests that vaccination with rds/CL/16 (H11) produces the same neutralizing response as its homologous antigen against the cnt/NL/15 (H11) PV in vitro. Mice vaccinated with both H13 antigens showed very little neutralization against the H11 PV, with only two mice of the lgl/NJ/17(H13) group and one mouse from the rbg/MN/17 (H13) group showing neutralization though IC_50_ values that were closer to the negative control group (Figure 9b, 1st panel). There was no neutralization of the H11 PV by mice vaccinated with H16 antigens, mgl/SCA/18 (H16) and bhg/NL/16 (H16), suggesting that this antigen did not elicit significant responses to epitopes that are common to both the H11 and H16 IAV strains.

When sera were tested against lgl/NJ/17 (H13) (Figure 9b, 2nd panel), mice vaccinated with H11 antigens produced poor neutralizing responses. However, similar to what we observed in the H11 PV neutralization (Figure 9b, 1st panel), there was no significant difference between the IC_50_ values of groups vaccinated with the other H13, rbg/MN/17 (H13), to those vaccinated with the homologous lgl/NJ/17 (H13) against the lglNJ/17 (H13) PV (Figure 9b, 2nd panel). Surprisingly, cross subtype neutralizing activity was observed in sera from mice vaccinated with both H16 antigens with no significant difference between the IC_50_ values of these groups with those vaccinated with the homologous lgl/NJ/17 (H13) against the lgl/NJ/17 (H13) PV (Figure 9b, 2nd panel). It is of note here that low level background neutralization was observed in the sera of the negative control group against lgl/NJ/17 (H13) PV, this was not seen when this group was tested against the H11 or H16 PV, reasons for this are currently unclear.

Interestingly, all vaccination groups neutralized the mgl/SCA/18 (H16) PV (Figure 9b, 3rd panel). Vaccinations with the other H16 antigen (bhg/NL/16 (H16)) and both H13 antigens achieved IC_50_ titres that had no significant difference (n.s.) compared to the homologous mgl/SCA/18 (H16) vaccination against the mgl/SCA/18 (H16) PV (Figure 9b, 3rd panel). Neutralizing responses were also observed in the sera of mice immunized with H11 antigens, albeit not as strong as that of the homologous ones, nonetheless these responses were significantly different from the negative control mice sera (*p* < 0.05) (Figure 9b, 3rd panel).

In the IBV study, for groups tested against the B/Hong Kong/1973 (B/HK/1973) PV, a pre-split IBV (Figure 9c, 1st panel) (*n* = 5 for all groups), no neutralization was observed in sera from mice regardless of the antigen they had been inoculated with including the pre-split antigen B/Singapore/1972 (B/SG/1972). None of the groups showed responses that were significantly different from sera collected from mice in the negative control group (PBS), which, incidentally, was showing some background against this PV. Most of the mice that were vaccinated with any of the antigens tested failed to reach 50% neutralization against the pre-split PV (Figure 9c, 1st panel).

When groups were tested against B/Yam/1988 (B/Yam) (Figure 9c, 2nd panel) (*n* = 5), sera from mice vaccinated with B/SG/1972 (pre-split) did not show any neutralization, except for one outlier that was outside the upper extreme quartile. Neutralization was observed for sera from all other groups including, as expected, those vaccinated with the antigen from Yamagata-like lineage (B/PHK/13) (Figure 9b, 2nd panel). Nonetheless, vaccination employing these antigens did not produce strong neutralizing responses against the B/Yam PV, as responses showed no significant difference with the PBS group, with IC_50_ dilution values ranging from 0.1 to 100.

Results for groups tested against B/Bri/08 PV (Figure 9c, 3rd panel) were interesting; as sera from mice in all groups were able to neutralize this B/Vic PV. Sera from mice vaccinated with B/SG/1972 (pre-split) and the other B/Vic antigen, B/CO/17, achieved IC_50_ dilution values, which had no significant difference (n.s) compared to that vaccinated with the homologous B/Bri/08 (B/Vic) against the B/Bri/08 (B/Vic) PV (*p* > 0.05). Sera from mice vaccinated with antigens from the other lineage, B/PHK/13 (B/Yam), achieved IC_50_ titres that were higher than those observed for mice from the group vaccinated with homologous B/Bri/08 (B/Vic) antigen (* *p* < 0.05) against B/Bri/08 (B/Vic) PV. This suggests that vaccination with either pre-split, B/Yam or B/Vic lineage antigens produces a significant neutralizing response against this B/Victoria PV.

### 3.4. In Vitro Neutralization of HA Pseudotypes by HA-Stem Directed Monoclonal Antibodies

It is desirable to have antibodies that will elicit a broad, cross-subtype specific response in order to address a pandemic threat. The influenza pseudotype microneutralization (pMN) assay is highly sensitive and specific for detecting neutralizing antibodies against influenza viruses regardless of whether they are HA-head specific or are targeted against the HA stem, making it an excellent test of antibody functionality in vitro [13,47,57,84]. Several broadly reactive monoclonal antibodies have been developed for use in immunotherapy against influenza. Monoclonal antibody CR9114 binds to IBV from both lineages and additionally binds influenza A viruses from both group 1 and group 2 [85], and FI6 is a pan-influenza A neutralizing antibody [84]. Both CR9114 and FI6 bind to a highly conserved epitope in the HA stem [84,85] enabling them to broadly neutralize influenza viruses and providing protection against a lethal influenza challenge in vivo. Here, we show neutralization of representative IAV and IBV PV by both mAbs (Table 4).

The half-maximal inhibitory concentration (IC_50_) of both mAbs against the PV tested were determined. Dose response curves (Figure S1) were obtained by normalizing the RLU values against that of the pseudotype only controls corresponding to 0% neutralization and cell-only (no virus) controls corresponding to 100% neutralization. A non-linear regression (curve fit) analysis on the normalized data using a log (inhibitor) versus normalized response variable slope equation to compute for the IC_50_ values was then carried out. The IC_50_ values for CR9114 against all IAV PV tested were in the range of 0.3–120 ng/mL (Table 4). The IC_50_ values for FI6 were more varied, with a range of 0.02–60 ng/mL (Table 4).

Both CR9114 and FI6 effectively neutralized key Group I Influenza A subtypes, H1, H2, H3, H5, H7, and H9 in vitro (Table 4, Supplementary Figure S1). These representative IAV subtypes were previously detected in the human population, including A(H1N1), A(H2N2), and A(H3N2), strains of which have previously caused global pandemics. Both mAbs were also able to neutralize all influenza PV representative strains from known avian subtypes for both the IAV Group I (H6, H8, H11, H12, H13, and H16) and Group II (H4, H10, H14, and H15) (Supplementary Figure S1). Notably, bat influenza H17 and H18 were also potently neutralized by CR9114 and FI6 (Table 4). In contrast, CR9114 and FI6 showed no neutralization activity against any of the influenza B strains tested. This correlates with the previous findings of CR9114 being unable to neutralize influenza B viruses in vitro as tested using the classic microneutralization and hemagglutination inhibition assays [85]. Some neutralization activity can be seen for CR9114 against B/Phuket/3073/2013, a B/Yamagata-like virus, at the highest concentration tested (1 µg/mL), but there was no dose–response established indicating no true neutralization occurred. As FI6 is only expected to neutralize influenza A viruses, we did not test it against IBV PV.

## 4. Discussion

Influenza infection contributes annually to morbidity and mortality in humans and in wild and domesticated animals worldwide even with vaccination programs already in place. There is additionally the ever-present threat of a pandemic brought about by novel influenza subtypes to which the population has no pre-existing immunity and of which seasonal vaccines may be unable to protect against. Protection provided by current seasonal influenza virus vaccines is generally limited and relies on predictive science. Ideally, vaccines should be rapidly generated upon the emergence of a novel threat and should be able to protect against both drifted and shifted strains, this is the goal of a universal vaccine approach.

To aid in efforts to create a universal influenza vaccine and assist in pandemic preparedness, we created a comprehensive influenza hemagglutinin pseudotype library. This library enables assessment of responses in lower containment settings thus negating the requirement for BSL3 facilities that are commonly required when working with high-risk influenza subtypes. Using pseudotypes also negates the need to isolate live viruses from clinical material, a process that is expensive and can be technically challenging and can potentially reduce the genetic authenticity of the isolated virus through egg adaptation. Generation of influenza pseudotyped lentiviral vector particles by transient transfection was achieved by utilizing the packaging construct p8.91, which drives the expression of all viral proteins required in trans [62]. Viral cis-acting sequences are then introduced into the same cell, maintaining the separation of viral genes and cis-acting sequences during production. This prevents recombination, leading to the production of replication-defective particles able to specifically transduce target cells. This system has been extensively optimized by Naldini et al. to ensure that recombination does not occur and to assess for safety [62,63,67,86]. The HA genes are then expressed by heterologous plasmids. Once the HA sequence has been identified, this can be cloned into a suitable plasmid expression vector. Here we utilized pI.18, pEVAC, and phCMV but other plasmids could be employed and the amount of DNA required determined using the optimization described in this study. Addition of a luciferase reporter plasmid produces results that can be determined rapidly using a system, which has the potential to be upscaled to high throughput platforms. Additionally, PV can be stored at −80 °C for extended periods of time and as was shown with H5, can be lyophilized and stored for up to 4 weeks at 37 °C [87]. Lyophilization could expand the potential to investigate and respond to pandemics or other outbreaks from any subtype at speed and without the need for cold chain storage.

We also showed that our HA PV library is suitable for the investigation of neutralization of sera collected from different species including mice, bats, sheep, and chickens (Figure 5, Table 2). The pseudotype library was also effective for use with neutralizing reference antisera (Figure 5), and this is integral to the vaccine strain selection process for seasonal influenza vaccines. Laboratories around the world that are part of the World Health Organization global influenza surveillance and response system monitor the antigenic phenotypes of circulating viruses to select vaccine strains for upcoming influenza seasons. However, investigation of emerging strains that could cause pandemics is limited, as it is arduous to isolate and propagate the wildtype virus to test against. Our influenza pseudotype library can be employed to test protection offered by existing vaccines and antisera used in their selection, in this instance, as tools for surveillance and pandemic preparedness.

We conducted several preliminary immunogenicity trials with selected vaccine antigens to inform the design of pivotal trials and to provide possible initial evaluation of vaccine efficacy employing our IAV and IBV pseudotypes. Other screening tools employ assays that evaluate the presence of binding antibodies but are unable to determine if these antibodies are functionally useful within samples. A common screening tool is the enzyme linked immunosorbent assay (ELISA), which can measure the total antibodies (e.g., total IgG) that bind to selected antigens. However, only a proportion of the total antibodies detected will be capable of inhibiting viral infection and this should be heavily taken into consideration when deciding how to measure the humoral immune response. Alternatively, the immune response may be assessed by neutralization assays employing native virus or viral pseudotypes. The latter can be carried out at BSL2 and allow a rapid, reliable, safe, and easy assessment of humoral immune responses of vaccine antigens against influenza subtypes, which are difficult to isolate and propagate.

We were able to show that immunization with prospective vaccine antigens and subsequent collection of blood serum samples at appropriate time intervals can be used to evaluate immune responses that are relevant to dosing strategies going forward (Figure 6). The data generated could inform the appropriate periods between doses and the number of doses that could provide the optimal immune response. In our immune response monitoring study, we found that immunization with 50 µg of pEVAC HA four times in 2-week intervals, produced optimal titres after the 4th inoculation, with immune responses at 56 and 70 days post immunization showing no significant difference (Figure 6d). Additionally, for vaccines, it may also be useful to explore the shortest time frame within which doses may be completed without a detrimental effect on the final immune response. Our results indicate that by employing our immunization and dosing strategy, a 4th immunization with pEVAC HA is necessary to achieve maximal titres, as lower titres were achieved with only three compared to four immunizations (Figure 6). This could be extended in the future to explore prime boost regimens with alternative vectors or proteins.

We also ran investigative trials wherein all mice received the same pEVAC HA vaccine antigen and we performed additional testing using relevant representative PV strains belonging to the same subtype (Figure 7 and Figure 8). It has been previously demonstrated that virus strains, even those within the same subtype, have different neutralization susceptibilities to sera that have been identified as cross-neutralizing, suggesting factors such as availability of HA epitopes via exposure, glycoprotein shielding and their role in antigenic drift, and immune evasion may be in play [88,89,90,91]. Our findings provide an indication as to whether immunization with a particular strain of the subtype can neutralize drifted strains of the same subtype, which is very important for lasting vaccine efficacy and protection especially in the case of influenza. This additional testing can also provide an assessment of the robustness and breadth of the humoral immune responses elicited by the vaccine to avian and human strains of the same subtype in the case of IAV and can guide the vaccine strain or antigen selection in a vaccine to improve or maintain its protective effect.

We also looked at comparing the immune response against phylogenetically related subtypes to investigate cross-subtype neutralization that can be brought about by vaccination (Figure 9). Here, we also selected IAV strains that are not usually studied, H10, H11, H13, H15, and H16, together with IBV from both lineages. Data from immunization protocols using these HA subtypes are very limited and to our knowledge, our group is among the first to report immune responses as a result of vaccination in these subtypes. Findings may aid in the development of a vaccine for pandemic purposes or inform possible pre-existing immune responses in the population. Immune responses generated by a vaccination with an HA antigen against the homologous PV was successful in all groups tested (Figure 6, Figure 7, Figure 8 and Figure 9). These can be used as control groups for future vaccination experiments. We found that cross-subtype protection is rare and neutralizing responses are not as strong as the homologous antigen against the PV. Several vaccinations include that of an H7 antigen that showed neutralization against an H10 and H15 PV (Figure 9a, 1st panel), H13 and H16 antigens showing cross neutralization with each other (Figure 9b), and B/Yamagata-lineage vaccinated mice neutralizing B/Victoria-lineage PV (Figure 9c). It can be hypothesized that immunogenic epitopes on the head domain of these rare HA subtypes are not under strong immune pressure in their natural avian reservoir and are unlikely to undergo genetic changes due to this pressure, but are more likely to be influenced by stochastic effects that conserve antigenic site sequences [92,93]. This results in the preservation of important antigenic epitopes such as the membrane-distal receptor-binding site (RBS) on the HA head containing key highly conserved amino acids involved in receptor binding [94,95,96,97] and can partially explain cross-subtype neutralization seen in our vaccination results. Nevertheless, our pseudotype library has enabled us to test immune responses brought about by vaccination against a variety of IAV and IBV pseudotypes. This is a promising in vitro screening tool to guide preclinical studies.

In addition to vaccination and antiviral drugs [50], the use of recombinant monoclonal antibodies that are broadly neutralizing against influenza is a promising strategy to counter annual epidemics and pandemic threats especially in individuals with severe disease. These mAbs, several of which are already in clinical development, bind to functionally conserved epitopes such as those in the influenza hemagglutinin (HA) stem, thereby providing strain independent protection [47,84,85,98,99]. For a time, discovery of these broadly neutralizing antibodies has been hampered by the lack of assays to properly show neutralization afforded by these mAbs that is exclusive from hemagglutination inhibition of HA-head directed antibodies. Antibodies that target the HA stem do not inhibit hemagglutination inhibition [51] and are thought to neutralize influenza via other mechanisms. We successfully employed our pseudotype library to investigate the neutralizing activity of HA stem-directed mAbs CR9114 and FI6 against representative IAV and IBV PV available. For instance, we could confirm that CR9114, despite binding to IBV HA, does not neutralize IBV in vitro. This is an invaluable tool to test functionality of new immunotherapies against influenza in vitro.

## 5. Conclusions

Lessons from the past have shown us, that despite our efforts, we are still unprepared to mitigate the devastating loss of life and livelihood when the next influenza pandemic occurs. The data presented in this study demonstrated the utility, versatility, and ease of employing influenza hemagglutinin pseudotyped viruses in preclinical studies to further vaccine research using reference standards, improve vaccine antigen design, and to evaluate alternative therapies such as mAbs, against influenza.

This library is expanding as influenza continues to undergo antigenic changes to the HA protein. We believe it can be part of a toolbox of assays that can be made available to researchers and will be especially helpful for studies investigating alternative and innovative influenza vaccine targets. This method employs a system that has the potential to be high throughput, can be easily adapted to other reporters, and may be incorporated into large scale clinical trials and surveillance programs. The PV can also be further developed in an ELISA where it will display the HA trimer in its native form and can be used to distinguish HA stalk responses and quaternary epitopes. Lentiviral PV can be constructed to display other potential vaccine targets such as neuraminidase (NA) and, in the future, we plan to complete a full NA PV library to complement our HA library. Additionally, these PV could be used to observe glycosylation patterns and their influence on the neutralization of influenza. We believe that our influenza PV library will be an invaluable tool for research and would be impactful in the development of solutions against the changing face of influenza.

## Figures and Tables

**Table 1:** Amounts of Influenza HA transfection components.

Solutions/Plasmids	Amount
OptiMEM	100 µL
p8.91	250 ng
pCSFLW	375 ng
HA in pEVAC	10 ng (50 ng for HA18)
HA in pI.18	50–500 ng
HA in phCMV1	50–500 ng
Protease-encoding plasmid	2.5–500 ng
FuGENE^®^ HD	3 µL per µg of total plasmid DNA

**Table 2:** Details of reference antisera obtained from OIE, NIBSC, and APHA for strains of IAV (H1-16) and IBV (B/Yam and B/Vic).

HA Subtype	Antiserum Strain	Source
H1	A/duck/Italy/447/2005 (H1)	OIE
H2	A/duck/Germany/1215/1973 (H2)	OIE
H3	A/psittacine/Italy/2873/2000 (H3)	OIE
H4	A/cockatoo/England/1972 (H4)	OIE
H5	A/chicken/Scotland/1959 (H5)	APHA
H6	A/turkey/Canada/1965 (H6)	OIE
H7	A/Anhui/1/2013 (H7)	NIBSC
H8	A/turkey/Ontario/6118/1968 (H8)	OIE
H9	A/mallard/Italy/3817-34/2005 (H9)	OIE
H10	A/ostrich/South Africa/2001 (H10)	OIE
H11	A/duck/Memphis/546/1974 (H11)	OIE
H12	A/duck/Alberta/60/1976 (H12)	OIE
H13	A/gull/Maryland/704/1977 (H13)	OIE
H14	A/mallard/Gurjev/263/1982 (H14)	OIE
H15	A/shearwater/Australia/2576/1979 (H15)	OIE
H16	A/gull/Denmark/68110/2002 (H16)	OIE
H17	Polyclonal sera (BATS)	APHA
B/YAM	B/Phuket/3073/2013	NIBSC
B/VIC	B/Brisbane/60/2008	NIBSC

**Table 3:** Titres in relative luminescence units/mL (RLU/mL) of IAV and IBV hemagglutinin pseudotyped viruses as indicated in Figure 3. Protease utilized to achieve the highest titres is indicated. TMPRSS4 is abbreviated to T4 and TMPRSS2 to T2.

Group I IAV HA			Group II IAV HA		
HA Envelope	Titre (RLU/mL)	Protease	HA Envelope	Titre (RLU/mL)	Protease
H1	2.25 × 10^8^	T4	H3	5.39 × 10^10^	T2
H2	6.62 × 10^7^	T2	H4	6.12 × 10^9^	T4
H5	1.32 × 10^9^	*	H7	5.25 × 10^10^	*
H6	2.35 × 10^10^	T4	H10	2.68 × 10^10^	T4
H8	4.75 × 10^10^	T4	H14	2.92 × 10^10^	T4
H9	4.88 × 10^8^	T4	H15	5.16 × 10^10^	T4
H11	8.78 × 10^9^	T4	**IBV HA**		
H12	1.21 × 10^10^	T4	B pre-split	3.87 × 10^10^	HAT
H13	1.44 × 10^9^	T4	B/Vic-like	2.89 × 10^10^	HAT
H16	5.81 × 10^9^	T4	B/Yam-like	1.78 × 10^9^	HAT
H17	2.94 × 10^8^	HAT			
H18	5.33 × 10^7^	T4			

(*) indicates highly pathogenic avian influenza (HPAI) strains, which do not require protease for production.

**Table 4:** IC_50_ (half-maximal inhibitory concentration) values of CR9114 and FI6 against representative influenza PV in vitro. (-) indicates no neutralization. n.d. indicates the experiment was not done.

Pseudotype Virus (PV)		IC_50_ (ng/mL)	
Subtype	Strain	*CR9114*	*FI6*
**H1**	A/England/195/2009	3.63	13.25
**H2**	A/quail/Rhode Island/16-018622-1/2016	5.06	26.70
**H3**	A/ruddy turnstone/Delaware Bay/606/2017	51.62	9.83
**H4**	A/Calidris ruficollis/Hokkaido/12EY0172/2012	1.68	8.36
**H5**	A/gyrfalcon/Washington/41088-6/2014	10.74	60.15
**H6**	A/American wigeon/California/HS007A/2015	0.68	2.91
**H7**	A/Shanghai/02/2013	11.88	17.39
**H8**	A/mallard duck/Ohio/16OS0672/2016	0.71	0.23
**H9**	A/chicken/Israel/291417/2017	0.39	6.41
**H10**	A/mallard/Utah/D1802334/2018	1.26	0.57
**H11**	A/red shoveler/Chile/C14653/2016	120.90	0.02
**H12**	A/duck/Mongolia/850/2018	15.06	0.51
**H13**	A/laughing gull/New Jersey/UGAI17-2843/2017	31.97	52.05
**H14**	A/blue-winged Teal/Ohio/18OS1695/2018	0.58	0.06
**H15**	A/wedge-tailed shearwater/Western Australia/2576/1979	10.73	14.12
**H16**	A/black-headed gull/Netherlands/1/2016	45.41	55.50
**H17**	A/little yellow-shouldered bat/Guatemala/60/2010	0.54	0.34
**H18**	A/flat-faced bat/Peru/033/2010	0.26	3.14
**B**	B/Hong Kong/8/1973	-	n.d.
**B/Vic**	B/Victoria/1/1987	-	n.d.
**B/Yam**	B/Yamagata/16/1988	-	n.d.

## Data Availability

The data presented in this study are available on request from the corresponding author.

## Supplementary Materials

The following are available online at https://www.mdpi.com/article/10.3390/vaccines9070741/s1, Figure S1. Neutralization of representative IAV and IBV PV in vitro by CR9114 and FI6. Table S1. List of influenza hemagglutinin pseudotypes (PV) available at the Viral Pseudotype Unit, University of Kent. Table S2. Neutralization susceptibility reported as IC_50_ dilution values of pseudotype viruses to HA specific antisera.
