# Supplementary material for: Exploiting Pan Influenza A and Pan Influenza B Pseudotype Libraries for Efficient Vaccine Antigen Selection
Source: Vaccines (Basel). 2021 Jul 5;9(7):741. doi: 10.3390/vaccines9070741 (PMC8310092; doi:10.3390/vaccines9070741)
Supplement: Supplementary file 1 [file vaccines-09-00741-s001.zip › vaccines-1234751-supplementary.pdf]

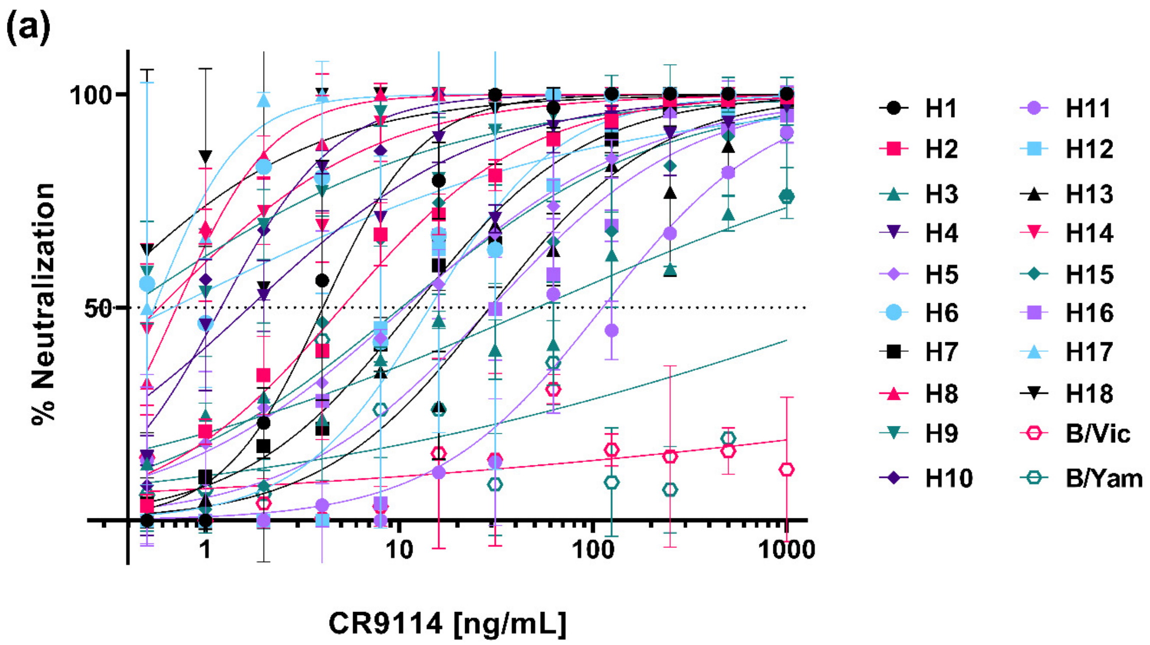

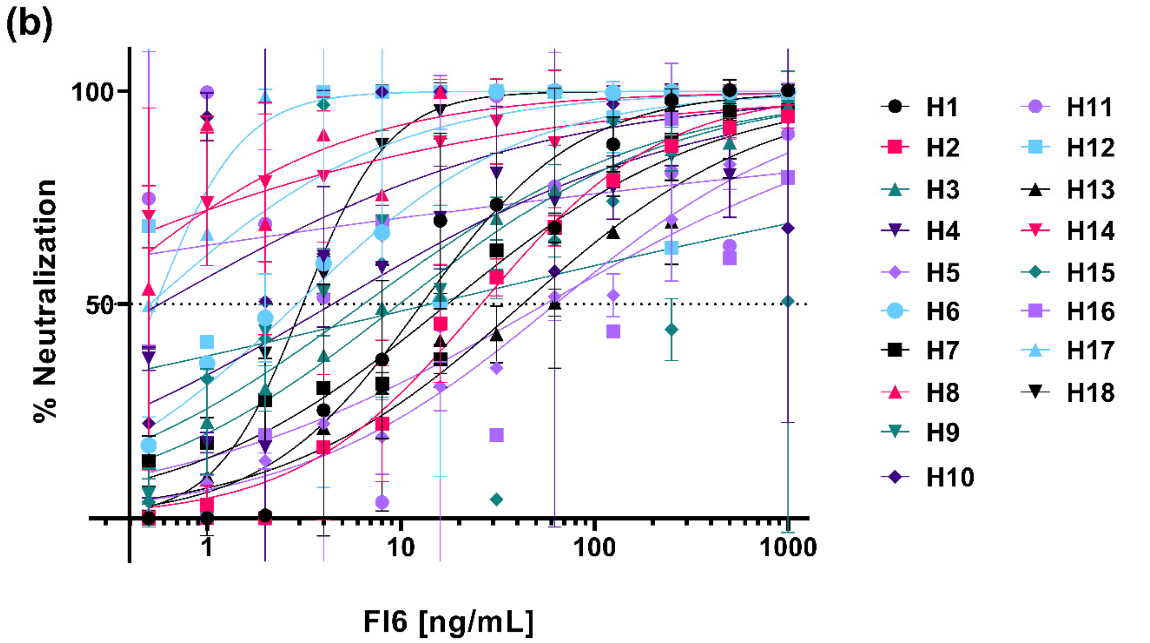

Figure S1. Neutralization of representative IAV and IBV PV *in vitro* by CR9114 and FI6. PV neutralization was measured by a luciferase reporter assay. **(a)** CR9114 and **(b)** FI6 were serially diluted two-fold from a starting concentration of 1000 ng/mL to 0.5 ng/mL against all pseudotypes. An input value of 1.0x10^{6} RLU of PV was then added to each well. For **(a)** and **(b)**, each point represents the mean and standard deviation of two replicates per dilution. IBV strains were not tested against FI6.

Table S1. List of influenza hemagglutinin pseudotypes (PV) available at the Viral Pseudotype Unit, University of Kent. Protease employed to achieve the highest pseudotype titers are indicated.

GROUP I INFLUENZA A HEMAGGLUTININ				
SUBTYPE	STRAIN	ACCESSION #	PLASMI D	PROTEASE
H1	A/South Carolina/1/1918	AF117241.1	phCMV1	TMPRSS4
	A/Puerto Rico/8/1934	AF389118.1	pI.18	TMPRSS4
	A/New Caledonia/20/1999	EU103824.1	phCMV1	TMPRSS4
	A/duck/Italy/1447/2005	HF563054.1	pI.18	TMPRSS4
	A/Solomon Islands/3/2006	EU124177.1	pI.18	TMPRSS4
	A/Brisbane/59/2007	CY163864.1	pI.18	TMPRSS4
	A/California/7/2009	CY121680.1	pI.18	TMPRSS4
	A/Texas/05/2009	GQ457487.1	pI.18	HAT
	A/England/195/2009	GQ166661.1	pEVAC	TMPRSS4
	A/Bolivia/559/2013	EPI466837	pEVAC	TMPRSS4
	A/swine/Guangxi/1/2013 2013_12_26_4	KJ725056	pEVAC	TMPRSS4
	A/Michigan/45/2015	EPI662594	pEVAC	TMPRSS4
	A/Slovenia/2903/2015	EPI768541	pEVAC	TMPRSS4
	A/Brisbane/02/2018	EPI1383389	pEVAC	TMPRSS4
	A/swine/Henan/SN10/2018 2018_02__4	MN416619	pEVAC	TMPRSS4
	A/swine/Beijing/0301/2018 2018_03__4	MN416589	pEVAC	TMPRSS4
H2	A/Korea/426/1968	CY125846.1	pI.18	HAT
	A/quail/Rhode Island/16-018622-1/2016 (H2)	KY272859	pEVAC	TMPRSS4
	A/duck/Germany/1215/1973	CY014710.1	pI.18	TMPRSS4
H5	A/Hong Kong/156/1997	AAC40508.1	pI.18	none
	A/Hong Kong/213/2003	ABP51977.1	pI.18	none
	A/Vietnam/1194/2004	ABP51976.1	pI.18	none
	A/Vietnam/1203/2004	AB51977.1	pI.18	none
	A/Indonesia/5/2005	ABW06108.1	pI.18	none
	A/turkey/Turkey/1/2005	ABD73284.1	pI.18	none
	A/Anhui/1/2005	ABD28180.1	pI.18	none

	A/whooper swan/Mongolia/244/2005	GU186700.1<br>ACZ36881.1	pEVAC<br>pI.18	none
	A/bar-headed goose/Qinghai/2005	BAE4815.1	pI.18	none
	A/Jwe/Hong Kong/1038/2006	ACJ26110.1A	pI.18	none
	A/chicken/Mexico/07/2007	KJ729343	pEVAC	none
	A/Egypt/2629-NAMRU/2007	ABM92273.1	pI.18	none
	A/chicken/Egypt 1709-01/2007	ACD64996.1	pI.18	none
	A/chicken/Egypt 1709-06/2008	ACD65000.1	pI.18	none
	A/gyrfalcon/Washington/41088-6/2014	KP307984	pI.18,<br>pEVAC	none<br>none
	A/mallard/Netherlands/41/2015	MF694083	pEVAC	none
H6	A/American wigeon/California/HS007A/2015	KY983173	pEVAC	TMPRSS4
	A/duck/Vietnam/HU9-455/2018	LC497121	pEVAC	TMPRSS4
H8	A/turkey/Ontario/6118/1968	CY014659.1	pI.18	TMPRSS4
	A/mallard duck/Netherlands/7/2015	MF682649	pEVAC	TMPRSS4
	A/mallard duck/Ohio/16OS0672/2016	MG280005	pEVAC	TMPRSS4
H9	A/Hong Kong/1073/1999	AJ404626.1	pI.18	TMPRSS4
	A/chicken/Israel/291417/2017	MH558944	pEVAC	TMPRSS4
H11	A/duck/Memphis/546/1974	AB292779	phCMV1	TMPRSS4
	A/common teal/Netherlands/1/2015	MF693986	pEVAC	TMPRSS4
	A/red shoveler/Chile/C14653/2016	MH134837	pEVAC	TMPRSS4
H12	A/duck/Alberta/60/1976	CY130078.1	phCMV1	HAT
	A/duck/Mongolia/850/2018	MK979051	pEVAC	TMPRSS4
	A/Northern Shoveler/Nevada/D1516557/2015	MK928236	pEVAC	TMPRSS4
H13	A/laughing gull/New Jersey/UGAI17-2843/2017	MH068343	pEVAC	TMPRSS4
	A/ring-billed gull/Minnesota/OPMNAI0816/2017	MH763859	pEVAC	TMPRSS4

H16	A/black-headed gull/Sweden/2/1999	AY684888.1	phCMV1	HAT
	A/black-headed gull/Netherlands/1/2016	MF694134	pEVAC	TMPRSS4
	A/Mew Gull/Southcentral Alaska/18MB01898/2018	MN210308	pEVAC	TMPRSS4
H17	A/Little shouldered bat/Guatemala/060/2011	CY103892.1	pI.18	HAT
H18	A/flat-faced bat/Peru/33/2010	CY125945	pEVAC	T4
**GROUP II INFLUENZA A HEMAGGLUTININ**				
**SUBTYPE**	**STRAIN**	**ACCESSION #**	**PLASMID**	**PROTEASE**
H3	A/Texas/50/2012	KC892952.1	pI.18	TMPRSS4
	A/Udorn/307/1972	DQ508929.1	pI.18	TMPRSS2
	A/California/7/2004	CY114373.1	pEVAC	HAT
	A/Wisconsin/67/2005	CY034116.1	pEVAC	HAT
	A/Japan/WRAIR1059P/2008		pEVAC	HAT
	A/Switzerland/9715293/2013	EPI814528	pEVAC	HAT
	A/New Caledonia/71/2014	EPI551570	pEVAC	HAT
	A/duck/Quang Ninh/220/2014	LC053492	pEVAC	HAT
	A/ruddy turnstone/Delaware Bay/606/2017	MH135712	pEVAC	TMPRSS2
	A/Kansas/14/2017	Vaccine strain, 3c3.A	pEVAC	HAT
	A/Switzerland/8060/2017	EPI1326015	pEVAC	TMPRSS4
	A/South Australia/34/2019	EPI1607117	pEVAC	HAT
H4	A/duck/Czechoslovakia/1956	D90302.1	phCMV1	TMPRSS2
	A/green-winged teal/California/K218/2005	CY045351	pEVAC	TMPRSS2
	A/Calidris ruficollis/Hokkaido/12EY0172/2012	LC467224	pEVAC	TMPRSS2
H7	A/FPV/Rostock/1934	AAA43150	phCMV1	none
	A/chicken/Pakistan/34668/1995	CY035831	pI.18	none

	A/chicken/Italy/1082/1999	CY022677	pI.18	TMPRSS2
	A/chicken/Italy/13474/1999	AJ491720	pI.18	none
	A/chicken/Netherlands/1/2003	AAR02640.1	pI.18	none
	A/chicken/Netherlands/219/2003	AAR02640.1	pI.18	none
	A/Shanghai/2/2013	KF021597 EPI448936	pI.18, pEVAC	TMPRSS4
	A/northern pintail duck/California/UCD1582/2016	MH251202	pEVAC	TMPRSS4
	A/duck/Viet Nam/HU10-64/2018	MK629228	pEVAC	TMPRSS4
	A/Anhui/1/2013	CY187618.1	pEVAC	TMPRSS4
H10	A/duck/Bangladesh/24268/2015	MH071504	pEVAC	TMPRSS4
	A/mallard/Utah/D1802334/2018	MK995817	pEVAC	TMPRSS4
	A/chicken/Germany/N49	CY014671.1	pEVAC	TMPRSS4
H14	A/mallard/Astrakhan/263/1982	AB289335.1 CY014604	phCMV1 pEVAC	TMPRSS4
	A/blue-winged Teal/Ohio/18OS1695/2018	MN431050	pEVAC	TMPRSS4
H15	A/shearwater/West Australia/2576/1979	CY130102.1 CY006010	phCMV1 pEVAC	TMPRSS4
	A/duck/Bangladesh/24697/2015	KY635719	pEVAC	TMPRSS4
**INFLUENZA B**				
**SUBTYPE**	**STRAIN**	**ACCESSION #**	**PLASMID**	**PROTEASE**
B	B/Hong Kong/8/1973	K00425	phCMV1	HAT
	B/Victoria/2/1987	FJ766840	phCMV1	HAT
	B/Yamagata/16/1988	CY018765.1	phCMV1	HAT
	B/Florida/4/2006	EU515992	phCMV1	HAT
	B/Bangladesh/3333/2007	CY115255.1	pI.18	HAT
	B/Brisbane/60/2008	KX058884.1 EPI753679	pI.18 pEVAC	HAT
	B/Phuket/3073/2013		pEVAC	HAT
	B/Colorado/06/2017		pEVAC	HAT

	B/Washington/2/2019	EPI1368874	pEVAC	HAT

Table S2. Neutralization susceptibility reported as IC_{50} dilution values of pseudotype viruses to HA specific antisera. IC_{50} dilution values reported below were calculated using GraphPad PRISM 8.12 from the dose response curves shown Figure 5.

	HA subtype	Estimated IC_{50} dilution
**Human-associated IAV** (Figure 5a)	H1 (A/Br/18)	14521
	H1 (A/En/09)	5011
	H2	2818
	H3	416
	H5	489
	H7	1659
	H9	3090
**Swine-associated IAV** (Figure 5b)	H1 A/Swine/Henan	Not calculated from curve
	H1 A/Swine/Guangxi	7943
	H1 A/Swine/Bejing	7762
**Avian-associated IAV** (Figure 5c)	H4 (A/GWT)	15135
	H4 (A/CR)	10471
	H6	Not calculated from curve
	H8	34673
	H10	3890
	H11	17378
	H12	Not calculated from curve
	H13	912
	H14	131825
	H15	5495
	H16	2344
**Bat Sera**	H17	724
	H17	537
	H17	645
**IBV**	B/Phuket (YAM)	Not calculated from curve
	B/Yamagata (YAM)	5754
	B/Washington (VIC)	5370
	B/Brisbane (VIC)	Not calculated from curve
	B/Colorado (VIC)	14454
	B/Victoria (VIC)	38904
